# Supplementary figures and images for: TBX5 R264K acts as a modifier to develop dilated cardiomyopathy in mice independently of T-box pathway
Source: PLoS One. 2020 Apr 1;15(4):e0227393. doi: 10.1371/journal.pone.0227393 (PMC7112173; doi:10.1371/journal.pone.0227393)

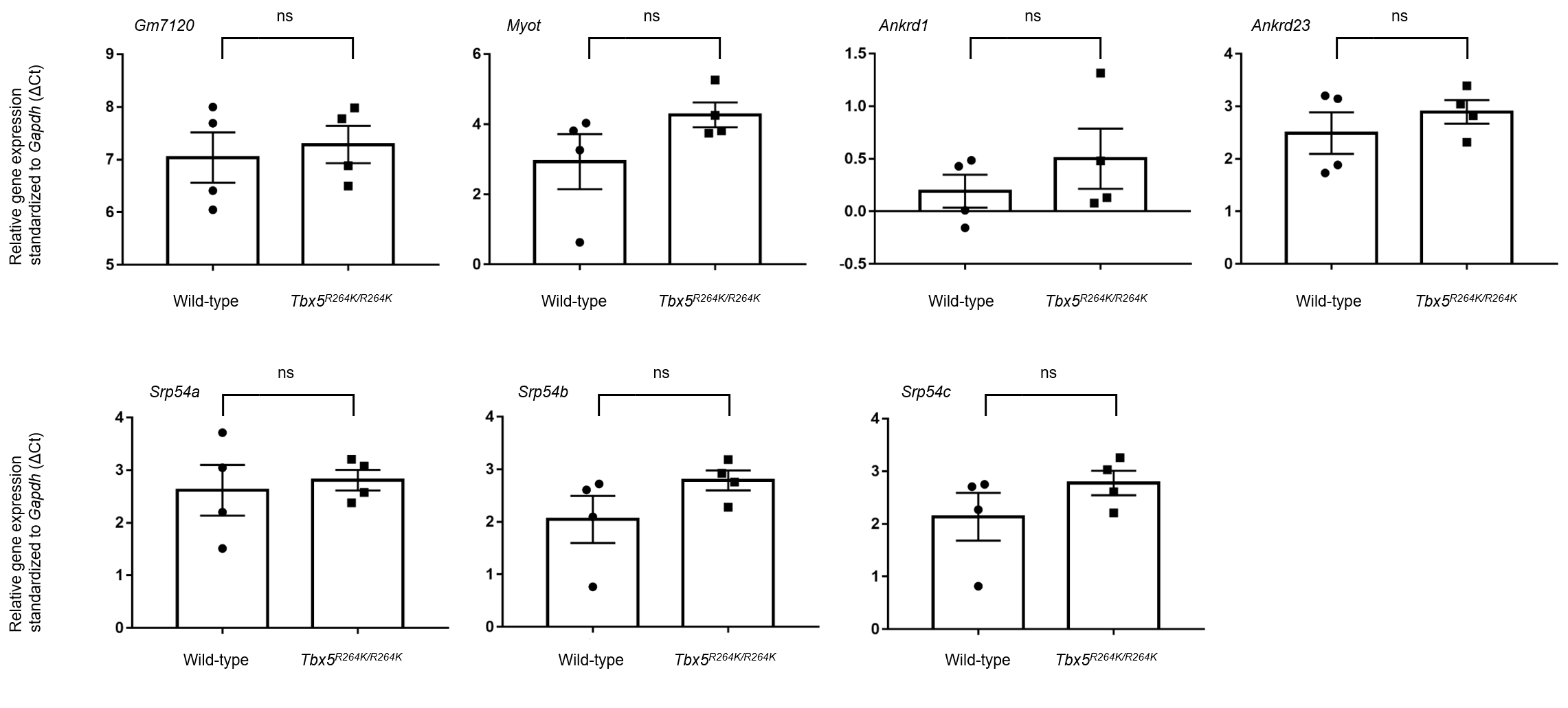

Supplement: S1 Fig — (TIF) [file pone.0227393.s001.tif]

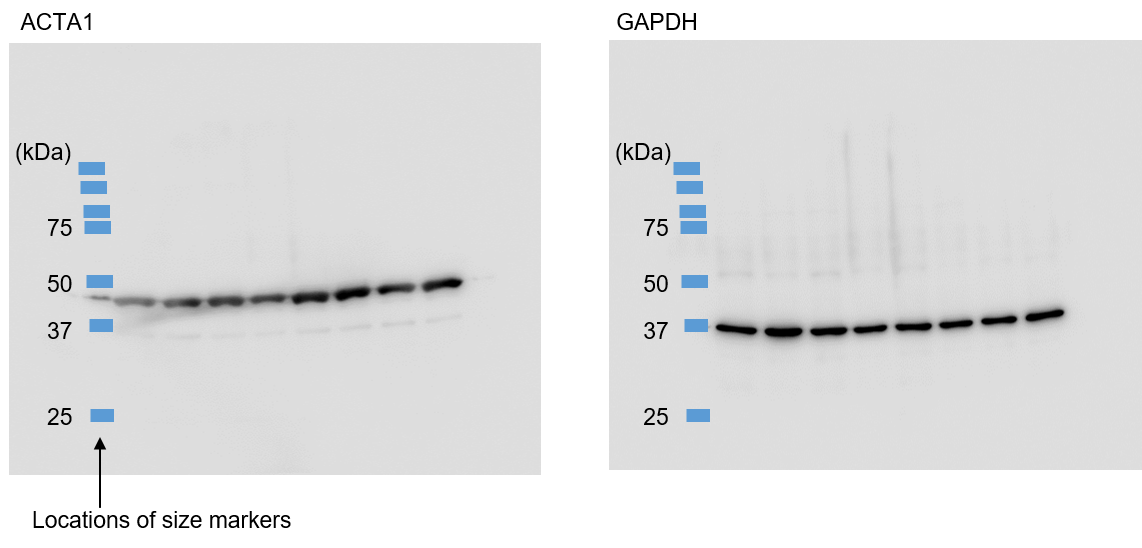

Supplement: S2 Fig — (TIF) [file pone.0227393.s002.tif]

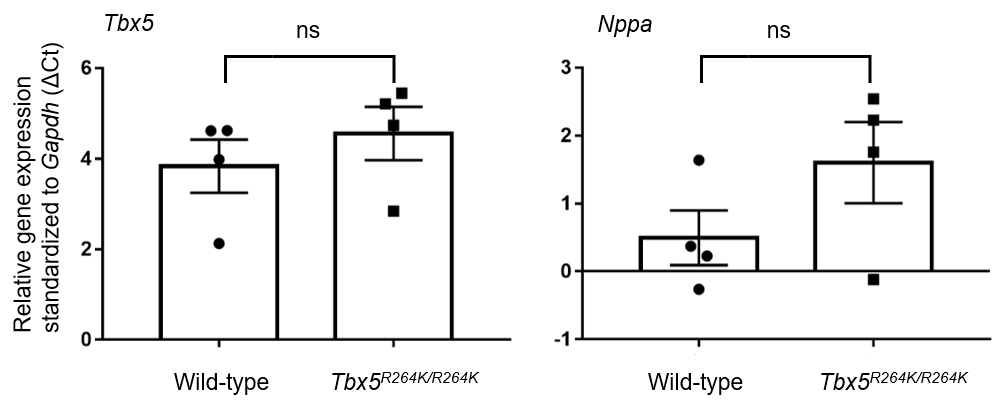

Supplement: S3 Fig — (TIF) [file pone.0227393.s003.tif]

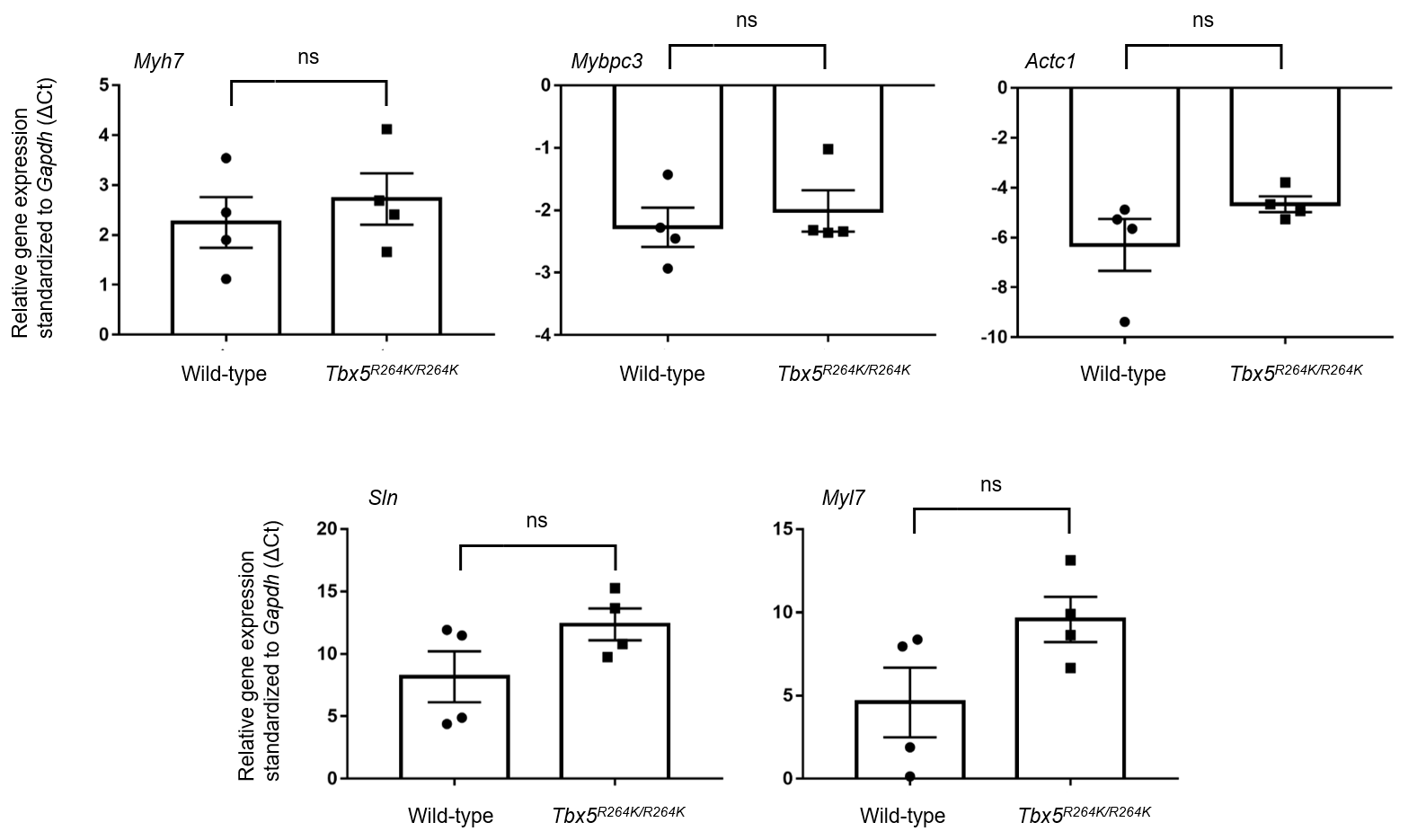

Supplement: S4 Fig — (TIF) [file pone.0227393.s004.tif]

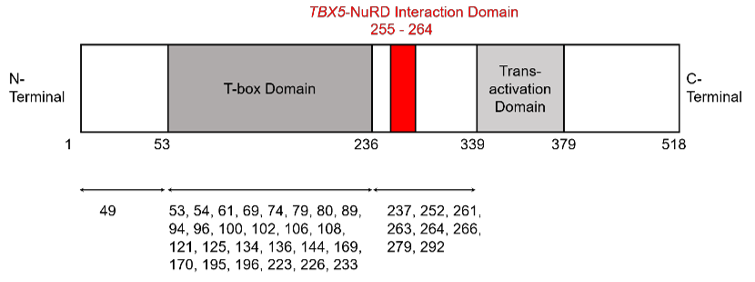

Supplement: S5 Fig — The numbers below indicate the positions of previously reported amino acid substitutions associated with congenital heart disease (35 variants in all). The variants described in this study, R264K, is located within the NuRD Interaction Domain. (TIF) [file pone.0227393.s005.tif]
