# Supplementary material for: TBX5 R264K acts as a modifier to develop dilated cardiomyopathy in mice independently of T-box pathway
Source: PLoS One. 2020 Apr 1;15(4):e0227393. doi: 10.1371/journal.pone.0227393 (PMC7112173; doi:10.1371/journal.pone.0227393)
Supplement: S1 File — (PDF) [file pone.0227393.s006.pdf]

S File.

Detailed Methods

Preparation of Cas9 mRNA, sgRNA and ssODN

The plasmid expressing hCas9 (pCAG-T3-hCas-pA) was a gift from Wataru Fujii and Kunihiro Naito (Addgene plasmid # 48625) [1], and was modified by replacement of the T3 promoter with the SP6 promoter (pCAG-SP6-hCas9-pA). The pCAG-SP6-hCas9-pA was linearized with *Sph*I, transcribed with mMESSAGE mMACHINE SP6 Ultra kit (Thermo Fisher Scientific, Waltham, MA, USA). gRNA was designed to target exon 8 of mouse T-box transcription factor 5 (*Tbx5*: 5'-TGGTTCCCAGGAGCACAGTGAGG-3'). The sgRNA expression plasmid was transcribed *in vitro* using PrecisionX Cas9 SmartNuclease RNA System (System Biosciences, Palo Alto, CA, USA). The synthesized RNAs were purified with RNeasy Mini kit (Qiagen, Valencia, CA, USA). The synthesized single stranded oligonucleotide (ssODN: 5'-CTTTGTCTTCTTCCTTTCAGTAAAGAGTATCCTGTGGTTCCCAGGAGCACAGTGAaGCACAAAGTCACCTCCAACCACAGCCCCTTCAGCAGCGAGACCCGAGCTCTCTC-3'; induced mutation was small caption) was purchased from Eurofins Genomics. Cas9 mRNA or protein/sgRNA/ssODN for microinjection was suspended in $T_{10}E_{0.1}$ buffer at 30 or 30/60/100 ng/uL.

Production of Cas9 protein

The coding sequence of Cas9 from pX362 [2] and synthetic oligonucleotides encoding V5 and histidine tags (Eurofins Genomics) were cloned into pET-11 (Merck Millipore) to yield the Cas9 expression vector, pET-11d-Cas9-VH. Cultures of Escherichia Coli RossettaGAMI (Novagen) transformed with the pET-11d-Cas9-VH were incubated for 7 hours in the presence of 1mM IPTG at 25 °C to induce the expression of the recombinant protein. The cells were lysed in Hanks balanced salt solution (HBSS) containing 0.5 % Triton X-100 and the Cas9 protein was purified using Talon affinity resin (Clonthech) followed by gel filtration with Superdex 200 (GE Healthcare) and dialyzed against Opti-MEM (Thermo Fisher Scientific).

Collection of embryos

Mouse pronuclear-stage embryos were obtained using an *in vitro* fertilization technique. C57BL/6N female mice at 4 weeks of age were superovulated by an intraperitoneal injection of IASe (0.1 mL IAS and 3.75 IU eCG, CARD HyperOva; Kyudo, Saga, Japan)

followed by hCG injection (7.5 IU, Gonatropin; Aska Pharmaceutical, Tokyo, Japan) 48 h later. Superovulated mice were euthanized at 15-16 h after hCG injection. Cumulus-oocyte complexes were collected from oviduct ampulla in HTF medium (ARK Resource, Kumamoto, Japan) covered with mineral oil (Sigma-Aldrich, St. Louis, MO, USA). They were inseminated with sperm from C57BL/6N male mice >10 weeks of age after pre-incubation in HTF medium for 1.5 h, and then were incubated at 37 ºC in 5 % CO2 and 95 % humidified air. After 3 h of incubation, the oocytes were washed four times with HTF medium. The pronuclear-stage embryos were cryopreserved 6.5 h after insemination. At later time points, the cryopreserved embryos were thawed by PB1 containing 0.25M sucrose (ARK Resource, Kumamoto, Japan). The freeze-thawed embryos were cultured in KSOM medium (ARK Resource, Kumamoto, Japan) before microinjection.

Microinjection

The pronuclear-stage embryos were transferred into M2 medium (Sigma-Aldrich, St. Louis, MO, USA) covered with mineral oil. Microinjection was performed on the stage of an inverted microscope (Axiovert-35; Zeiss, Jena, Germany) equipped with micromanipulators (Eppendorf, Hauppauge, NY, USA). Cas9 mRNA or protein, sgRNA and ssODN mixtures were injected into the pronuclei of each embryo. The injected embryos were incubated in KSOM medium covered with mineral oil until they were transferred into the oviducts of pseudopregnant ICR female mice at the two-cell stage.

Genotyping

Genomic DNA was extracted from mouse tail biopsies and subjected to PCR amplification targeting the genomic sequences around the target site of the gRNA using KOD-plus-Neo DNA polymerase (Toyobo, Osaka, Japan) and the primers: 5’-AGGCTCAGCAAGGAGGTGAA-3’ and 5’-CATGGCAGCGAGCAGTAAGG-3’. The PCR products were directly sequenced using the BigDye terminator v3.1 cycle sequencing mix (Thermo Fisher Scientific, Waltham, MA, USA).

Supporting Reference

1. Fujii W, Kawasaki K, Sugiura K, Naito K. Efficient generation of large-scale genome-modified mice using gRNA and CAS9 endonuclease. *Nucleic Acids Res. 2013;* 41:e187.
2. Ito T, Hayashida M, Kobayashi S, Muto N, Hayashi A, Yoshimura T, Mori H. Serine racemase is involved in D -aspartate biosynthesis. *J Biochem. 2016;* 160: 345–353.
