# Supplementary material for: TBX5 R264K acts as a modifier to develop dilated cardiomyopathy in mice independently of T-box pathway
Source: PLoS One. 2020 Apr 1;15(4):e0227393. doi: 10.1371/journal.pone.0227393 (PMC7112173; doi:10.1371/journal.pone.0227393)
Supplement: S1 Table — (PDF) [file pone.0227393.s007.pdf]

S1 Table. List of the genes associated with inherited cardiac disease that were analyzed by NGS.

ABCC9, ACTC1, ACTN2 AKAP9, ANK2, BAG3, BMPR1A, CACNA1C, CACNB2, CALR3, CAPN3, CAV3, DES, DMD, DSC2, DSG2, DSP, ELN, EMD, GAA, GATA4, GLA, GPD1L, HCN4, JUP, KCNE1, KCNE2, KCNE3, KCNH2, KCNJ2, KCNQ1, KRAS, LAMP2, LDB3, LMNA, MIB1, MYBPC3, MYH11, MYH6, MYH7, MYL2, MYL3, MYLK, MYOZ2, NKX2-5, NRAS, PKP2, PLN, PRDM16, PRKAG2, PTPN11, RAF1, RPS7, RYR2, SCN1B, SCN3B, SCN4B, SCN5A, SGCD, SMAD3, SNTA1, SOS1, STARD3, TAZ, TBX5, TGFBR1, TGFBR2, TMEM43, TNNC1, TNNI3, TNNT2, TPM1, VCL
