# Supplementary material for: TBX5 R264K acts as a modifier to develop dilated cardiomyopathy in mice independently of T-box pathway
Source: PLoS One. 2020 Apr 1;15(4):e0227393. doi: 10.1371/journal.pone.0227393 (PMC7112173; doi:10.1371/journal.pone.0227393)
Supplement: S2 Table — (PDF) [file pone.0227393.s008.pdf]

S2 Table. Grading scale for reactive fibrosis and replacement fibrosis.

**Grading scale for fibrosis**
Reactive Fibrosis
0=Negative
1=Perivascular
2=Perivascular with extension to interstitium
a. focal
b. diffuse
3=Encircling of individual myocytes
a. without perivascular fibrosis
b. with perivascular fibrosis
Replacement Fibrosis
0=Negative
1=Minimal foci
2=Occasional foci and small scars
3=Extensive scarring
