# Supplementary material for: TBX5 R264K acts as a modifier to develop dilated cardiomyopathy in mice independently of T-box pathway
Source: PLoS One. 2020 Apr 1;15(4):e0227393. doi: 10.1371/journal.pone.0227393 (PMC7112173; doi:10.1371/journal.pone.0227393)
Supplement: S3 Table — (PDF) [file pone.0227393.s009.pdf]

S3 Table. PCR primer sequences (5′to 3′) used to real time RT-PCR.

Genes	Sequences (forward primer)	Sequences (reverse primer)
Gapdh	acagtccatgccatcactgc	taggaacacggaaggccatg
Acta1	gtcctgcaagtgaacaagcc	caggatggagccaccgatc
Gm7120	agcgggatttttagcgtctgt	gaaggaaaggtcttcgcgga
Ankrd1	tcgactcttgatgaccttcgg	ttgctttggttccactctgc
Ankrd23	ggcaccacaaagcaacgaaa	ctgccagtctcgagccag
Myot	cagaatgctgctgctgtgtt	acttgtgatgtgggaacgtg
Srp54a	cacagatggcaaaattaaaccaaca	actgccgcatcattgactga
Srp54b	gccaaaatgatggacccacg	cctttcatgttgccagcagc
Srp54c	gccaaaatgatggacccacg	cctttcatgttgccagcagc
Tbx5	cccacctaacccatacccac	acggcttcttataggggtgc
Nppa	tcgggggtaggattgacagg	cagaatcgactgccttttcctc
Myh7	ctccatctctgacaacgcct	tagtgttgacagtcttcccagc
Mybpc3	ctgctcccactgtggtctg	ccactcctcatcagcaccag
Sln	tggaggtggagagactgagg	tccagaaggacttgtgattgca
Actc1	tgaagatcctcactgagcgc	tcaaaatccagggcgacgta
Myl7	aagccatcctgagtgccttc	gaacttgtctgcctgggtca
