# Supplementary material for: TBX5 R264K acts as a modifier to develop dilated cardiomyopathy in mice independently of T-box pathway
Source: PLoS One. 2020 Apr 1;15(4):e0227393. doi: 10.1371/journal.pone.0227393 (PMC7112173; doi:10.1371/journal.pone.0227393)
Supplement: S4 Table — (PDF) [file pone.0227393.s010.pdf]

S4 Table. Variant identified in the patients

gene	protein	coding	dbsnp	FATHMM	SIFT	Polyphen-2	Align GVGD	Mutation Taster	Provean	CADD	Minor allele frequency		
											gnomAD	gnomAD (East Asian)	HGVD
TBX5	p.Arg264Lys	c.791G>A	rs201071418	Damaging score; -1.57	Damaging score; 0	Probably damaging score; 0.991	C25	Disease causing	Tolerated score: -0.533	26.7	0.0001168	0.001654	0.0075
