# Supplementary material for: TBX5 R264K acts as a modifier to develop dilated cardiomyopathy in mice independently of T-box pathway
Source: PLoS One. 2020 Apr 1;15(4):e0227393. doi: 10.1371/journal.pone.0227393 (PMC7112173; doi:10.1371/journal.pone.0227393)
Supplement: S5 Table — (PDF) [file pone.0227393.s011.pdf]

S5 Table. ***In silico*** **predictive algorithms used in the study [3].**

Category	Basis	Name	Website	Prediction Threshold
Missense prediction	Evolutionary conservation	FATHMM	http://fathmm.biocompute.org.uk	<-1.5 Damaging<br>>-1.5 Tolerated
		SIFT	http://sift.jcvi.org	<0.05 Deleterious<br>>0.05 Tolerated
Missense prediction	Protein structure/function and evolutionary conservation	Align GVGD	http://agvgd.iarc.fr/agvgd_input.php	≧C15 Probably Damaging
		Mutation Taster	http://www.mutationtaster.org	Disease causing
		Polyphen-2	http://genetics.bwh.harvard.edu/pph2	0.85 to 1 Probably Damage<br>0.15 to 0.85 Possibly Damage
Missense and insertion/deletions prediction	Alignment and measurement of similarity between variant sequence and protein sequence homolog	PROVEAN	http://provean.jcvi.org/index.php	<-2.5 Deleterious<br>>-2.5 Neutral
Missense and insertion/deletions prediction	Contrasts annotations of fixed/nearly fixed derived alleles in humans with simulated variants	CADD	http://cadd.gs.washington.edu	≧20 1% most deleterious<br>≧30 0.1% most deleterious

Supporting Reference

3. Richards S, Aziz N, Bale S, Bick D, Das S, Gastier-Foster J, Grody WW, Hegde M, Lyon E, Spector E, Voelkerding K, Rehm HL. Standards and guidelines for the interpretation of sequence variants: a joint consensus recommendation of the American College of Medical Genetics and Genomics and the association for molecular autopsy. *Genet Med.* 2015; 17: 405–423.
