# Supplementary material for: TBX5 R264K acts as a modifier to develop dilated cardiomyopathy in mice independently of T-box pathway
Source: PLoS One. 2020 Apr 1;15(4):e0227393. doi: 10.1371/journal.pone.0227393 (PMC7112173; doi:10.1371/journal.pone.0227393)
Supplement: S6 Table — (PDF) [file pone.0227393.s012.pdf]

S6 Table. Characteristics and echocardiographic data in wild-type and *Tbx5*$^{R264K/R264K}$ mice in mature to middle age. *P* value *<0.05.

	Mature to middle age wild-type (n=7)	Mature to middle age *Tbx5*$^{R264K/R264K}$ (n=6)
male	3	3
BW (g)	33.5±5.7	33.6±5.1
HR (/min)	432.8±29.3	435.3±18.8
LVDd (mm)	4.21±0.42	4.57±0.41
LVDs (mm)	2.87±0.29	3.38±0.44*
AWD (mm)	0.83±0.16	0.73±0.1
PWD (mm)	0.9±0.22	0.7±0.09
FS (%)	31.8±2.6	26.1±4.0*
E (cm/s)	63.3±16.4	50.7±15.8
A (cm/s)	42.7±11.2	31.6±15
heart weight (mg)	155.3±17.4	161.2±9.3
HW/BW	4.69±0.57	4.89±0.87
