# Supplementary material for: TBX5 R264K acts as a modifier to develop dilated cardiomyopathy in mice independently of T-box pathway
Source: PLoS One. 2020 Apr 1;15(4):e0227393. doi: 10.1371/journal.pone.0227393 (PMC7112173; doi:10.1371/journal.pone.0227393)
Supplement: S7 Table — (PDF) [file pone.0227393.s013.pdf]

S7 Table. Electrocardiogram data comparing *Tbx5*$^{R264K/+}$ and wild type mice

		RR Interval (s)	Heart Rate (BPM)	PR Interval (s)	P Duration (s)	QRS Interval (s)	QT Interval (s)	QTc (s)	JT Interval (s)	Tpeak Tend Interval (s)	P Amplitude (mV)	Q Amplitude (mV)	R Amplitude (mV)	S Amplitude (mV)	ST Height (mV)	T Amplitude (mV)
Hetero-Male	39-8-10	0.0994	603.5700	0.0368	0.0081	0.0176	0.0424	0.1346	0.0248	0.0225	0.1092	−0.5417	0.5026	−0.6665	0.0356	0.0401
	39-8-11	0.1464	409.8100	0.0379	0.0084	0.0171	0.0416	0.1087	0.0245	0.0174	0.0504	−0.0357	0.7263	−0.7097	−0.0156	−0.0377
	39-8-13	0.1173	511.3900	0.0364	0.0067	0.0105	0.0356	0.1040	0.0251	0.0215	0.0558	−0.0029	0.6569	−0.4462	−0.0217	−0.0215
	39-8-15	0.1313	456.8400	0.0412	0.0101	0.0177	0.0426	0.1175	0.0248	0.0151	0.0445	−0.0218	0.3369	−0.3736	−0.0019	−0.0228
	39-8-19	0.1298	462.1600	0.0373	0.0089	0.0167	0.0439	0.1219	0.0272	0.0191	0.1038	−0.1948	1.9601	−0.8218	−0.0376	−0.0567
Hetero-Female	39-8-16	0.1285	466.9200	0.0392	0.0081	0.0170	0.0401	0.1119	0.0231	0.0154	0.0743	−0.1051	1.1012	−0.8270	−0.0126	−0.0525
	39-8-17	0.1423	421.6200	0.0470	0.0100	0.0153	0.0407	0.1080	0.0254	0.0118	0.0684	−0.0118	0.3979	−0.3910	0.0088	−0.0474
	39-8-18	0.1359	441.5800	0.0419	0.0086	0.0153	0.0510	0.1384	0.0357	0.0291	0.0708	−0.0719	1.4835	−0.5222	−0.0703	−0.0946
	39-8-30	0.1346	445.6900	0.0400	0.0079	0.0106	0.0412	0.1124	0.0306	0.0197	0.1052	0.0067	0.9389	−0.4926	−0.0180	−0.0711
	39-8-31	0.1247	481.3700	0.0391	0.0103	0.0172	0.0411	0.1165	0.0240	0.0164	0.0817	−0.1109	0.9310	−0.4990	−0.0317	−0.0477
WT-Male	39-8-20	0.1554	386.3300	0.0376	0.0073	0.0137	0.0438	0.1113	0.0301	0.0212	0.0515	0.0049	0.5444	−0.3036	−0.0573	−0.0359
	39-8-21	0.1373	437.0000	0.0369	0.0113	0.0105	0.0502	0.1355	0.0398	0.0260	0.0974	−0.0068	0.6604	−0.1843	0.0912	−0.0633
	39-8-22	0.1684	356.3900	0.0397	0.0105	0.0167	0.0516	0.1258	0.0349	0.0293	0.0708	−0.0531	1.4498	−0.5332	−0.0952	−0.1212
	39-8-28	0.1108	541.7200	0.0339	0.0036	0.0178	0.0292	0.0877	0.0114	0.0083	0.0676	−0.3589	0.6416	−0.3882	0.0469	0.0469
	39-8-29	0.1394	430.3200	0.0425	0.0071	0.0186	0.0390	0.1043	0.0204	0.0172	0.0697	0.0026	0.6829	−0.4224	−0.0397	−0.0447
WT-Female	39-8-32	0.1459	411.1400	0.0395	0.0075	0.0185	0.0407	0.1066	0.0222	0.0160	0.0655	−0.1207	0.9207	−0.5266	−0.0224	−0.0472
	39-8-33	0.1454	412.6900	0.0428	0.0104	0.0155	0.0495	0.1298	0.0340	0.0267	0.0586	−0.0755	0.9019	−0.4348	−0.0272	−0.0603
	39-8-34	0.1269	472.9700	0.0402	0.0078	0.0175	0.0426	0.1195	0.0251	0.0179	0.0596	−0.0719	0.5628	−0.2304	−0.0145	−0.0289
	39-8-35	0.1382	434.0400	0.0413	0.0074	0.0167	0.0421	0.1134	0.0254	0.0180	0.0798	−0.0586	0.8116	−0.4834	−0.0246	−0.0538
	39-8-36	0.1400	428.5600	0.0372	0.0100	0.0174	0.0439	0.1175	0.0265	0.0202	0.0569	−0.1689	1.1684	−0.3903	−0.0502	−0.0664

		RR Interval (s)	Heart Rate (BPM)	PR Interval (s)	P Duration (s)	QRS Interval (s)	QT Interval (s)	QTc (s)	JT Interval (s)	Tpeak Tend Interval (s)	P Amplitude (mV)	Q Amplitude (mV)	R Amplitude (mV)	S Amplitude (mV)	ST Height (mV)	T Amplitude (mV)
Hetero-Male		0.1249	488.7540	0.0379	0.0084	0.0159	0.0412	0.1173	0.0253	0.0191	0.0727	−0.1594	0.8366	−0.6036	−0.0083	−0.0197
WT-Male		0.1422	430.3520	0.0381	0.0080	0.0154	0.0428	0.1129	0.0273	0.0204	0.0714	−0.0823	0.7958	−0.3663	−0.0108	−0.0436
Hetero-Female		0.1332	451.4360	0.0415	0.0090	0.0151	0.0428	0.1174	0.0278	0.0185	0.0801	−0.0586	0.9705	−0.5464	−0.0248	−0.0627
WT-Female		0.1393	431.8800	0.0402	0.0086	0.0171	0.0438	0.1173	0.0267	0.0198	0.0641	−0.0991	0.8731	−0.4131	−0.0278	−0.0513
WT		0.1408	431.1160	0.0392	0.0083	0.0163	0.0433	0.1151	0.0270	0.0201	0.0677	−0.0907	0.8345	−0.3897	−0.0193	−0.0475
mut		0.1290	470.0950	0.0397	0.0087	0.0155	0.0420	0.1174	0.0265	0.0188	0.0764	−0.1090	0.9035	−0.5750	−0.0165	−0.0412
	P value of Ttest	0.085	0.114	0.692	0.606	0.510	0.613	0.696	0.878	0.607	0.320	0.773	0.713	0.011	0.884	0.722
