# Supplementary material for: TBX5 R264K acts as a modifier to develop dilated cardiomyopathy in mice independently of T-box pathway
Source: PLoS One. 2020 Apr 1;15(4):e0227393. doi: 10.1371/journal.pone.0227393 (PMC7112173; doi:10.1371/journal.pone.0227393)
Supplement: S8 Table — (PDF) [file pone.0227393.s014.pdf]

S8 Table. Terms about biological processes inferred by g:Profiler

source	term_name	term_id	adjusted_p_value	negative_log10_of_adjusted_p_value	term_size	query_size	intersection_size	effective_domain_size	intersections
GO:BP	response to muscle stretch	GO:0035994	0.015927463	1.797853406	19	7	2	21207	ANKRD1,ANKRD23
GO:BP	SRP-dependent cotranslational protein targeting to membrane	GO:0006614	0.015927463	1.797853406	19	7	2	21207	SRP54C,SRP54A
GO:BP	cotranslational protein targeting to membrane	GO:0006613	0.017694398	1.752164208	20	7	2	21207	SRP54C,SRP54A
GO:BP	response to mechanical stimulus	GO:0009612	0.038920733	1.409818993	223	7	3	21207	ANKRD1, ANKRD23,ACTA1
