# Supplementary material for: TBX5 R264K acts as a modifier to develop dilated cardiomyopathy in mice independently of T-box pathway
Source: PLoS One. 2020 Apr 1;15(4):e0227393. doi: 10.1371/journal.pone.0227393 (PMC7112173; doi:10.1371/journal.pone.0227393)
Supplement: Attachment — Submitted filename: PONE-D-19-34630_reviewer-1 comments.docx [file pone.0227393.s015.docx]

**Dear Editor,**

**Authors have performed a very laborious work, considering the characterization of a TBX5 SNP in a transgenic murine model, following a population study. The current manuscript is performed by the application of state-of-art techniques, that efficiently support the data. Some minor and major comments should be considered for the improvement of the manuscript in favour of the authors and the readers.**

**Minor** **Comments**

1. Authors are suggested to change the title from “TBX5 R264K induces chronic heart failure not via T-box pathway” to “TBX5 R264K induces chronic cardiomyopathy independently of T-box pathway”, so that it is more appealing to the reader.
2. Please avoid the use of the term heart failure in your in vivo experiments, as a NYHA scoring should be performed to claim it. Please change the term to cardiomyopathy, when referring to the murine models.
3. Table 2B: Please correct Ds to LVDs.
4. Figure 5, considering the Western Blot analysis of ACTA1 is not self explanatory. Proper labelling of the WB images is missing, as well as the molecular weight of the identified targets and the labelling of the samples. The authors are suggested to change it accordingly.
5. The resolution of the figures is poor, which creates an inconvenience for the reader. Authors are suggested to provide a higher image resolution to the given figures. Moreover, concerning the column plots, authors are suggested to use another software, rather than Microsoft Office Excel, in order to export more appealing plots and figures.

**Major Comments**

1. Authors state to have used transgenic mice of 6-9 months old as an aging model. However, according the literature, mice of that age correspond to human adults around 40 years of age. In order to consider a mouse aged, the minimum age of 18 months should be reached. Authors are advised either to correct the statement, or to present mortality data justifying the selection of the specific age for their murine model.
2. Hematoxylin-Eosin staining seems to be poorly performed as nuclei (blue) are not visible in the histology images. Please refine your staining, by improving your H&E protocol and by washing your specimens with PBS for the removal of blood (as visible in Fig. 1A lower panel). Moreover, considering that authors are commenting on intracardiac fibrosis in their transgenic mice, maybe another more specific staining should be applied such as Sirius Red or Masson Trichrome. By the use of these staining techniques the visualization of collagen deposition will be more straight-forward.
3. Column graphs are not presented in a sufficient manner. Individual values, showing the distribution and the number of the biological replicates should be present in the graphs (i.e. scatter plotted column graphs) and statistical analysis (i.e. asterisks) should be presented, deducing the statistically significantly differences between the groups.
4. The use of isoproterenol is not sufficiently justified. Transgenic mice, already present cardiac contractility deficits (e.g. decreased FS%) in the absence of the β-agonist. Therefore, the use of isoproterenol should be better discussed in the Discussion section, considering its transcriptional value in the experiments.
5. Authors shown that the given SNP in the TBX5 leads to a downregulation of ACTA-1 in the myocardium. Concerning that ACTA-1 is a highly important protein in the cellular cytoskeleton, authors are suggested to perform electron microscopical evaluation of the cardiomyocytes’ structure in the transgenic mice. Therefore, a more detailed representation of the effect of TBX5 SNP on cardiomyocytes’ architecture will be available.
6. If applicable, it would be interesting if the authors could confirm the results from their transgenic murine model in human cardiac biopsies, following simple RT-PCR or Western Blot experiments. Thus, the translational value of the current manuscript will be increased.
